# Strategies to Overcome Biological Barriers Associated with Pulmonary Drug Delivery

**DOI:** 10.3390/pharmaceutics14020302

**Published:** 2022-01-27

**Authors:** Adam J. Plaunt, Tam L. Nguyen, Michel R. Corboz, Vladimir S. Malinin, David C. Cipolla

**Affiliations:** Insmed Incorporated, Bridgewater, NJ 08807, USA; Tam.nguyen@insmed.com (T.L.N.); Michel.corboz@insmed.com (M.R.C.); Vladimir.malinin@insmed.com (V.S.M.); David.cipolla@insmed.com (D.C.C.)

**Keywords:** inhaled drug delivery, prodrug, liposome formulation

## Abstract

While the inhalation route has been used for millennia for pharmacologic effect, the biological barriers to treating lung disease created real challenges for the pharmaceutical industry until sophisticated device and formulation technologies emerged over the past fifty years. There are now several inhaled device technologies that enable delivery of therapeutics at high efficiency to the lung and avoid excessive deposition in the oropharyngeal region. Chemistry and formulation technologies have also emerged to prolong retention of drug at the active site by overcoming degradation and clearance mechanisms, or by reducing the rate of systemic absorption. These technologies have also been utilized to improve tolerability or to facilitate uptake within cells when there are intracellular targets. This paper describes the biological barriers and provides recent examples utilizing formulation technologies or drug chemistry modifications to overcome those barriers.

## 1. Introduction

Pulmonary delivery of therapeutics is now generally accepted as an ideal strategy to deliver an effective amount of drug to the airways to treat diseases including asthma, chronic obstructive pulmonary disease (COPD), cystic fibrosis (CF) and pulmonary arterial hypertension (PAH), among others [1]. Many of the early inhaled therapies were repositioned after initially being administered by the oral or injectable routes [2]. This change in delivery route was initiated in many cases to avoid systemic side effects and improve targeting to the lung allowing delivery of higher doses that improved efficacy. The inhalation route was not the original choice due to the inconvenience, poor efficiency, variability in delivered dose and lack of portability of the early generation of aerosol delivery technologies [3]. However, tremendous innovation has occurred in the past fifty years and product developers can now choose among portable dry powder inhalers (DPIs), metered dose inhalers (MDIs), soft mist inhalers (SMIs) and nebulizers that provide reproducible and efficient delivery to the lung.

The anatomy of the respiratory tract represents the first biological barrier to effective drug delivery to the conducting airways and deeper bronchopulmonary segments. For successful delivery to the lung, the inhaled aerosol must avoid deposition in the oropharyngeal region. As aerosol droplets or particles are inhaled, their momentum can lead to deviation from a bending air flow path, resulting in aerosol impaction on the surfaces of the mouth or throat and a reduction in the dose to the lung [4,5]. Aerosol devices delivering particles with smaller aerodynamic diameters, which can be achieved by a combination of lower density or smaller geometric size, can more easily avoid aerosol impaction in the oropharyngeal region. Additionally, slower patient inhalation flow rates can also reduce oropharyngeal deposition and some device technologies facilitate this paradigm [6]. Furthermore, the aerosol should be delivered in an aerosol volume that can be fully inhaled with each breath, or else delivery efficiency may be compromised. Synchronization of aerosol generation with initiation of inhalation is a feature of some “smart” inhalation systems [7].

In summary, innovations in inhaler device technologies have addressed the first biological hurdle, which is to minimize oropharyngeal deposition, resulting in a reproducible dose of drug to the lung. The criticality of the reproducibility of lung delivery depends on the drug and the indication. Those indications with narrow therapeutic windows may require delivery systems with exceptional control over the emitted dose and how the aerosol is generated and inhaled. While avoiding oropharyngeal deposition is the first biological barrier, there remain other biological barriers summarized below. Each barrier may need to be considered for each new therapeutic opportunity to understand its significance as an impediment to achieving the desired treatment paradigm. In this paper, we provide examples of how these barriers can be overcome using formulation technologies or modifying the chemistry of the compound.

## 2. Biological Barriers

**Figure 1:**
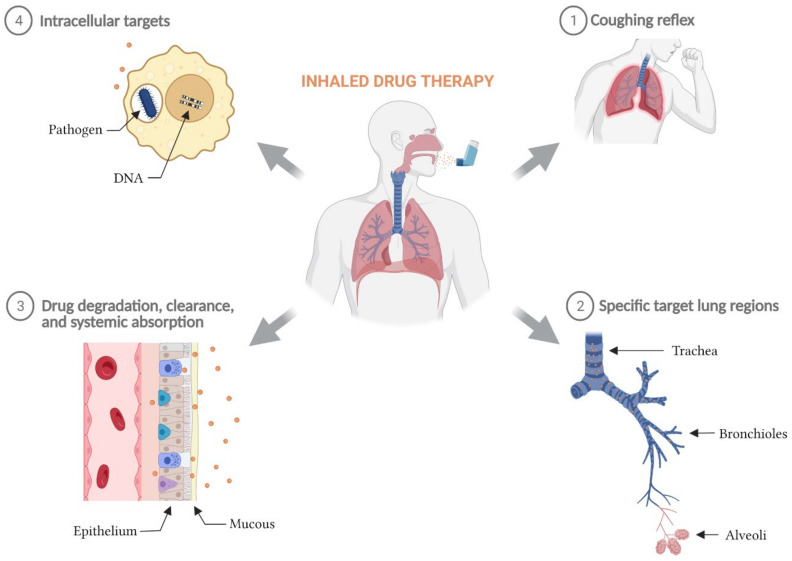
Biological barriers to inhaled drug therapy. The major biological barriers to a successful inhaled drug therapy include: (**1**) post-inhalation coughing reflex, (**2**) low delivery efficiency to specific target lung regions, (**3**) rapid elimination of inhaled active molecules from the lung via degradation, clearance, and/or systemic absorption, and (**4**) the inability for inhaled therapies with intracellular targets to sufficiently penetrate cellular phospholipid membranes and maintain therapeutic concentrations intracellularly.

The major biological barriers to achieving successful inhaled drug therapy have been documented [8], are summarized in Figure 1 and include:

- Avoiding the cough reflex so that the complete aerosol dose can be inhaled and deposited at its pulmonary target site;
- Achieving efficient delivery of the aerosol to the target region within the lung. For example, to ensure that the therapeutic dose reaches the deeper structures of the lung, the aerosol must be able to pass through the trachea-bronchial airways of the respiratory tract with limited deposition;
- Interacting with the airway surface liquid and mucus to access the cellular targets. Upon deposition in the lung fluid, the drug particle may need to be dissolved or released from a matrix, and for prodrugs, may need to be chemically converted to its active form before being able to diffuse to its target site to take effect. Physical barriers can impede that process;
- Overcoming systemic absorption, degradation or clearance of the active molecule to provide drug concentrations that remain within the therapeutic window until the next administration event. When these functions are relatively rapid, therapeutic levels of the drug can be reduced to ineffective levels prior to the next inhalation event;
- Accessing intracellular targets. Many targets may reside within cells including macrophages, and the cellular membrane presents a barrier to delivery of drug within the cells.

### 2.1. Avoiding the Cough Reflex

Inhaled therapies that result in coughing during the inhalation event may lead not only to incomplete inhalation of the therapeutic, but also to greater upper airway deposition or exhalation of the aerosol that is in transit during inhalation. Cough during an inhalation event may reduce the efficacy of that treatment event and if recurring, would generally lead to non-adherence. Post-inhalation coughing may not physically hinder inhaled drug delivery, but it may be a barrier to patient treatment adherence. Some inhaled drugs or formulations may lead to a coughing bout by triggering two types of afferent nerves: extrapulmonary Widdicombe cough receptors and/or the intrapulmonary bronchopulmonary C-fibers. The former type is sensitive to extreme pH [9,10,11] and mechanical stimuli while the latter is sensitive to prostaglandin E2, bradykinin, and a variety of environmental irritants [12,13]. Extreme local osmolarity of the airway surface liquid in the vicinity of dissolving inhaled particles can also activate the afferent nerves and generate a coughing reflex [10,14]. Post-inhalation cough frequency varies with sex and age of patients. Sensitivity to chemical stimuli are increased in female and pediatric patients [15,16] while the frequency of cough reflex is significantly lower in elderly patients than in young patients [17,18]. The cough reflex can be overcome by reducing the rate of particle dissolution on the airway surface or by utilizing other formulation strategies that minimize changes in the proton concentration or osmolality of the epithelial lining fluid [19].

### 2.2. Depositing in the Target Region within the Lung

The respiratory tract can be divided into 3 main regions: the upper airways or the oropharyngeal region, the lower airways or the trachea-bronchial region, and the gas exchange or alveolar region [20]. The upper airways consist of the nose, the nasal cavity, the pharynx, and the larynx, while the lower airways comprise the trachea, bronchi, bronchioles and terminal bronchioles. Gas exchange occurs in the alveoli at the end of the terminal bronchioles. From the trachea to the alveoli, there are approximately 23 diverging and asymmetric divisions. As inhaled air travels along the complex respiratory tract, many particles in the airstream are removed by inertial impaction at airway bifurcations [5]. At the end of inhalation, the air flow is reversed. Inhaled particles with smaller size or lower density have limited time to settle to their target sites unless a breath hold maneuver is incorporated at the end of the inhalation. Particles still air-borne at the end of inhalation are then driven out of the lung and some of them land on the respiratory tract walls along the way [5]. Therefore, while employing inhaled air to deliver drugs to the lung is a sensible approach, targeting exclusively the trachea-bronchial or the alveolar regions is extremely difficult [5]. Utilizing more rapid inhalations and larger aerosol particles (e.g., 6 µm) will increase trachea-bronchial deposition over alveolar deposition, although at the expense of greater deposition in the oropharynx. Targeting deposition of inhaled drugs to the alveolar regions can be increased by reducing inhalation flow rate and aerosol size (e.g., to 1–3 µm), and can be further increased by a breath hold to facilitate deposition by sedimentation. Several studies using radio-labelled particles clearly demonstrated the impact of changes in particle size and inhalation flow rate on drug deposition [21,22]. Moreover, in diseased lungs, the challenge for effective inhaled drug delivery is even more significant due to airway constrictions [23], excessive mucous [24], and/or edema [25].

### 2.3. Interacting with the Airway Surface Fluid and Overcoming Systemic Absorption, Degradation or Clearance

The surface liquid of the lower airways is composed of 2 layers: a mucous layer with varying thickness [26,27] and a periciliary liquid layer on the airway epithelium. The mucous layer is a gel-like, entangled network of heavily and heterogeneously glycosylated mucins. The diverse carbohydrate chains on mucins allow the mucous layer to interact with and trap a wide range of particles and microorganisms [28] in the inhaled air [29]. The watery periciliary liquid layer is found beneath the mucous layer where the epithelial cilia reside. Synchronized beatings of the epithelial cilia transport mucous and periciliary liquid toward the mouth [30]. Particles trapped by the airway mucus or in the periciliary liquid can be cleared from the lung in a relatively short time frame [31] and thus reduce the active drug concentration, potentially necessitating more frequent dosing. The gas exchange regions are not lined with mucous, making them better target sites for inhaled drugs from that perspective. However, the alveolar epithelial-endothelial layer is thin, and passive transport of small molecular weight drugs into the systemic circulation can be rapid [32] providing only transient drug retaining capacity unless facilitated by a sustained release formulation. In diseased lungs, the mucous of the lower airways can be overexpressed and/or more thickened [26,33], blocking access for inhaled drugs to the target tissues beneath.

The epithelial layer lies immediately beneath the airway surface liquid with its apical surface facing the air space and its basal surface in contact with fluid-filled lung parenchyma. The epithelial layer restricts passages of ions and larger solutes to maintain lung fluid balance. Therefore, crossing the epithelial cell layer can be challenging for inhaled hydrophilic drugs targeting the lung parenchyma. Receptor-mediated transcytosis mechanisms have been identified in lung epithelial cells for some small peptides [34,35]. The efficiency of epithelial active transport depends on the lipophilicity of the molecule, the number of receptors, and the amount of consumed energy. For hydrophilic molecules, slow diffusion through the paracellular tight junction is the only option, and the opening of this tight junction limits the rate of transport for larger molecules [32]. Two distinct pathways through epithelial tight junctions have been identified—the pore pathway mediating passage of small solutes and the leak pathway mediating passage of macromolecules [36,37,38]. In lung alveolar epithelial cells, the leak pathway depends on the lung-specific tight junction protein claudin 18 [39]. The composition of the leak pathway is still under debate; however, it is known that the “tightness” of the epithelial tight junction varies greatly between the lung regions and is closely regulated by epithelial intracellular activities [38,40].

For people with lung diseases, the epithelial layer is subject to complex structural and functional changes. Infection can cause some component proteins of the tight junction to be upregulated while others are downregulated [41,42]. In asthma, COPD, CF, and idiopathic pulmonary fibrosis, epithelial cells undergo epithelial-to-mesenchymal transition and lose crucial proteins of the tight junctions [43,44]. Epithelial cell damage and massive death are common in *Pseudomonas aeruginosa* infection, acute lung injury, and acute respiratory distress syndrome [45,46,47].

### 2.4. Accessing Intracellular Targets

Another barrier for a pharmaceutical molecule to access an intracellular target is the phospholipid bilayer cell membrane. Intracellular treatment targets may include microbiological pathogens, receptors, and attenuation or correction of protein synthesis through the delivery of gene therapy, mRNA and siRNA delivery. Intracellular microbial pathogens often reside within lung alveolar macrophages due to active phagocytotic processes.

As essential components of innate immunity, macrophages play multiple roles in immune surveillance, defense against pathogens, and resolution of inflammation. Airspace macrophages (AMs) are readily retrievable from the airspaces via bronchoalveolar lavage (BAL) and have been well-studied [48]. The healthy human lung contains between 1.4 × 10^10^ and 2.3 × 10^10^ AMs, with over 97% present in the alveolar lumen [49,50] A similar number of macrophages are found in the lung interstitium, known as interstitial macrophages (IM), with 78% of all IMs being present in the alveolar septa [49].

Lung resident macrophages further polarize between the M1 state (classically activated macrophages) and the M2 state (alternatively activated macrophages). M1 macrophages play essential roles in host defense through expression of proinflammatory and antimicrobial signals while M2 macrophages help maintain tissue homeostasis and control inflammation through expression of anti-inflammatory cytokines [51,52].

Macrophages have been identified as important sites of infection by both viruses and bacteria for several diseases, including tuberculosis (TB) and non-tuberculous mycobacteria (NTM) lung disease. Microbial pathogens have developed diverse strategies to survive and hide from the host immune response inside the macrophages [53,54], making it difficult to eradicate these pathogens with traditionally formulated antibiotics. Some of these intracellular pathogens reside in the host’s cytosol while others reside in intracellular vacuoles. Thus, targeting of the intracellular pathogen populations within the macrophages in the lung presents a particular challenge for developing certain anti-infective lung therapeutics.

Most antibiotics do not easily penetrate cell membranes. Among therapeutics that have been shown to accumulate in macrophages are bedaquiline, oritavancin, and telavancin [55,56,57]. Novel vancomycin derivatives have also demonstrated the ability to target intracellular infections [58].

The most frequent strategy to improve the penetration of antibiotics into phagocytic cells is the use of carrier systems that deliver these drugs directly to the target cell. Delivery systems such as liposomes, nanoparticles, lipid systems, and conjugates enhance the therapeutic efficacy of antibiotics and antifungal agents in the treatment of infections caused by intracellular microorganisms [54,59,60].

## 3. Strategies to Overcome Biological Barriers

Through decades of effort, scientists, chemists, and engineers have developed hardware and technology solutions to overcome, and in some cases even capitalize on, the numerous barriers described above. As the relevance of these biological barriers differs by disease, strategies to overcome said barriers is also disease specific. In the context of pulmonary drug delivery, two main strategies are used to overcome these biological barriers: nanoparticle formulation and/or modification of a known active pharmaceutical ingredient (API) using a prodrug strategy. The final choice to use either or both strategies can be nuanced and is dependent on the therapeutic area.

Nanoparticle formulation strategies can be used to alter the way in which active compounds are presented within the body [61,62,63,64,65], including modification of their dissolution profiles [66,67,68,69,70], and some of these strategies have been adapted specifically for pulmonary delivery [71,72,73,74]. In some instances, nanoparticles can help overcome specific pharmaceutical development challenges including increased payload delivery, controlled-release kinetics to optimize pharmacokinetic (PK)/pharmacodynamic (PD), improvement of efficacy, and reduction of adverse events [64,75,76]. Similarly, a prodrug modification strategy can be used to alter a compound’s PK/PD profile to optimize performance following pulmonary administration. Prodrugs are chemical modifications of an active compound using a labile covalent attachment of a pro-moiety that alters key physical-chemical properties of the active compound, and thus drastically reduces its activity prior to cleavage of the pro-moiety [77,78]. By definition, prodrugs are inactive until the labile covalent bond is cleaved, and the active compound is released. The prodrug strategy carries some extra regulatory burden in that the pro-moiety must be biocompatible and the cleavage mechanism must be robust in all species studied. Furthermore, in the context of pulmonary drug delivery, the decision to use either strategy is impacted by multiple factors including the choice of the delivery device (i.e., DPI, MDI, nebulizer, or soft mist inhaler), the specific disease, and the potency of the active compound.

## 4. Inhaled Treprostinil Palmitil (TP) for the Treatment of Pulmonary Arterial Hypertension (PAH)

PAH is a progressive, life-threatening disease characterized by the constriction and remodeling of the pulmonary vasculature, leading to increased pulmonary arterial resistance and pulmonary arterial pressure [79,80,81,82,83,84] that may result in right heart failure and ultimately death [79,80,81,82,83,84,85,86,87]. Treatment of PAH often involves administration of prostacyclin analogs as pulmonary vasodilating agents. Although these therapies can be effective, most of them are unable to overcome a variety of barriers that make effective treatment difficult to achieve including tolerability issues, rapid absorption, and the requirement for frequent repeat administrations [79,85,86].

One of the most well-known treatments for PAH is a synthetic prostacyclin derivative called treprostinil (TRE). TRE therapies are available as an inhalation solution (Tyvaso), an oral tablet (Orenitram), and as a subcutaneous infusion (Remodulin). TRE acts as a vasodilator with excellent clinical efficacy but its short half-life requires either frequent administration or continuous infusion [87,88]. Frequent administration of TRE results in peak-trough cycles where high levels of TRE shortly after administration may cause patients to experience adverse events, and low levels of TRE prior to the next round of administration may lead to a lack of therapeutic efficacy. Furthermore, inhaled TRE presents tolerability issues with patients often experiencing cough and throat irritation [87,89]. Fortunately, there are ongoing clinical-stage efforts to develop improved, alternative prostacyclin derivative therapies using a variety of pulmonary delivery approaches including liposome encapsulation of iloprost [90,91,92] and TRE [93,94], nanoparticle formulation [95,96], advanced 3D printing techniques as a way to engineer uniform respirable particles [97,98], and prodrug modification [99].

Development of an inhaled prodrug can be challenging because the associated cleavage mechanism and release of the pro-moiety must be well controlled and not cause any undue toxicities. Therefore, selection of the prodrug chemistry and structure must be well thought out. In the case of TP, a hydrophobic prodrug of TRE, the carboxylic acid functional group from TRE is masked with an ester bond using a 16-carbon hydrophobic chain to form the prodrug. When delivered to the lungs, esterase enzymes that are ubiquitous in the lung microenvironment hydrolyze the ester bond releasing TRE, the active compound, and cetyl alcohol as an inert pro-moiety. Early in vitro experiments confirmed that the rate of hydrolysis for TRE prodrugs can be tuned based on the alkyl chain length of the pro-moiety [99].

Initial formulation efforts using TP focused on a solid lipid nanoparticle formulation, called treprostinil palmitil inhalation solution (TPIS) that was delivered via a nebulizer. This formulation was noteworthy because it relied on three separate strategies to overcome the biological barriers associated with TRE. Firstly, it used a hydrophobic prodrug that allowed for sustained release of TRE. Secondly, it capitalized on the use of solid lipid nanoparticles to aid in solubility (as a technique to deliver a hydrophobic drug in an aqueous media). And thirdly, by delivery of the formulation directly to the lungs using a nebulizer, it targeted the API to the area of interest in the body. Taken together, these strategies resulted in an extended release of TRE that maintains efficacy through 24 h with significantly lower plasma TRE levels, and provides a localized effect specific to the organ of interest, in this case the lungs [100]. More recently, TP has been reformulated for convenient delivery using a DPI using a formulation called Treprostinil Palmitil Inhalation Powder (TPIP), while maintaining the sustained release of TRE. The ability to re-formulate as a DPI is largely due to the high potency of this drug, requiring mere micrograms of material to achieve a therapeutic response.

**Figure 2:**
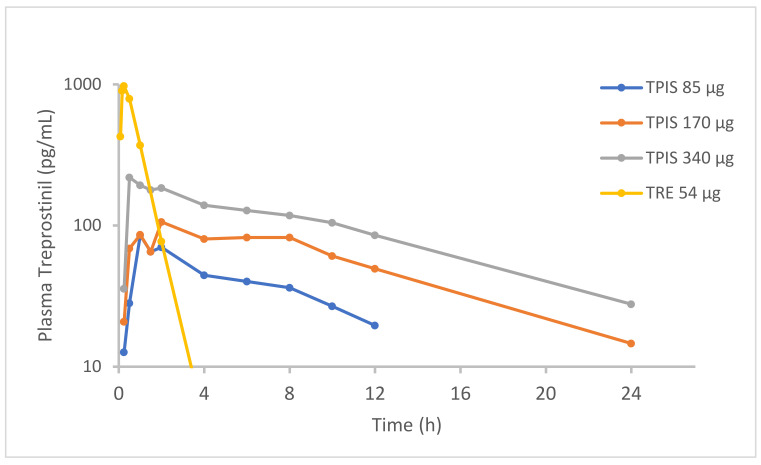
Pharmacokinetics (PK) of Treprostinil (TRE) in healthy volunteers after administration of Treprostinil Palmitil Inhalation Solution (TPIS) or nebulized TRE. Adapted from [104]. European Respiratory Society, 2016.

Indeed, in vivo PK data comparing TRE to TP confirms that administration of the prodrug results in meaningfully reduced peak plasma TRE concentrations and sustained release of TRE over 24 h [101,102,103]. Clinical studies with both TPIS and TPIP confirmed sustained release profiles of the TP formulation compared to inhaled TRE [100]. TPIS administered at a dose 85 µg (equivalent to TRE dose 54 µg) demonstrated prolonged TRE half-life of 6.4 h, compared to 0.50 h for inhaled TRE solution dosed at 54 µg (Figure 2). The peak plasma TRE concentration following TPIS administration was only 97.6 pg/mL which is roughly 10-fold lower than the peak plasma TRE concentration following a 54 µg dose of TRE (985 pg/mL). TPIS bioavailability was not notably affected, resulting in a systemic plasma TRE exposure (AUC0-24h) of 0.617 ng*h/mL, similar to 0.893 ng*h/mL after TRE inhalation. A similar PK profile was observed following administration of TPIP [manuscript submitted].

**Figure 3:**
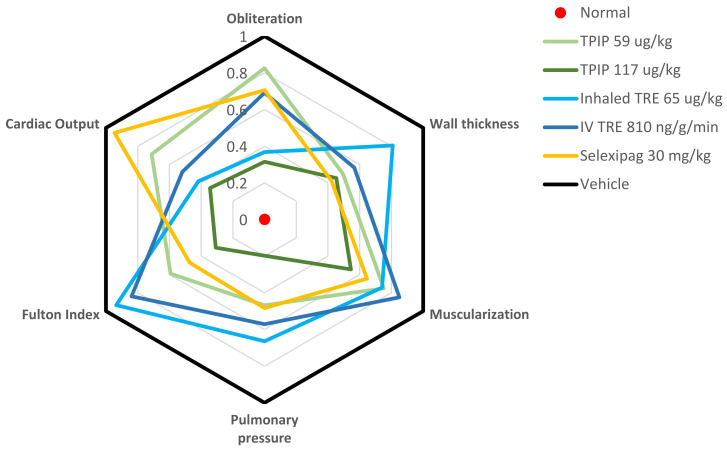
Effect of Treprostinil Palmitil Inhalation Powder (TPIP) in a Sugen/Hypoxia (Su/Hx) rat Pulmonary Arterial Hypertension (PAH) model. This spider graph visually depicts the various readouts from the Su/Hx model including indices of hemodynamics (pulmonary arterial pressure), right ventricular hypertrophy (Fulton index, cardiac output), and vascular remodeling (wall thickness, muscularization and obliteration). In the graph, the individual parameters are represented on separate axes radiating out from the center of the figure. Each parameter is normalized between the normal healthy state (a score of zero, at the center of the figure) and the vehicle control representing the injury after exposure to Su/Hx (a score of 1, at the periphery of the figure). The findings for each compound are depicted in various colors. In this type of figure, the closer that the lines are to the center, the more efficacy they demonstrate. Fulton Index = weight ratio of Right Ventricle/(Left Ventricle + Septum), Pulmonary Pressure = mean Pulmonary Arterial Pressure, Obliteration = percentage (%) of non-obliterated vessels, Wall thickness = Small vessel wall thickness, Muscularization = % of muscularized vessels, Cardiac Output = amount of blood pumped by the heart per minute. Adapted from [105]. American Thoracic Society, 2021.

When tested in a Sugen/Hypoxia (Su/Hx) rat model of PAH, and in comparison with (1) inhaled TRE, (2) intravenous TRE and (3) oral Selexipag, TPIP significantly out-performed the comparator agents [105]. TPIP was associated with a reduction in pulmonary pressure, a reduction in the percentage of muscularized vessels, and a reduction in the percentage of obliterated vessels (Figure 3). Similar results were also observed for the initial nebulizer formulation, TPIS [106]. TPIS demonstrated durable efficacy in vivo, showing a significant reduction in hypoxia-induced right ventricular pressure (RVPP) at plasma TRE concentrations much lower than that observed for infused TRE. In addition to PK/PD advantages, it is worth noting that TP formulations also result in reduced cough and tachyphylaxis relative to TRE in rodent models which could translate to improved patient tolerability via reduction in adverse events in the clinic [107,108]. Thus, for an inhaled prostanoid therapy, the prodrug strategy coupled with an inhaled dry powder format, may enable a once-daily therapy that overcomes the tolerability and rapid absorption barriers, and possibly provide improved efficacy if the remodeling observed in the Su/Hx model translates into the clinic.

## 5. Nebulized CL27c for the Treatment of Pulmonary Fibrosis

Pulmonary fibrosis (PF) is a progressive respiratory condition characterized by chronic fibrosis of the lung interstitial tissues that is associated with diminished lung function and a high mortality rate with limited treatment options [109]. The most common form of PF is idiopathic pulmonary fibrosis (IPF), meaning that the root cause of disease is unknown [110]. During the progression of IPF, alveolar epithelial cells become over-activated which results in accumulation of fibroblasts and myofibroblasts in addition to extensive matrix remodeling [111]. As part of this remodeling, the epithelium becomes scarred and develops a thickened alveolus wall that interferes with gas exchange reducing pulmonary function. Treatment for pulmonary fibrosis typically involves administration of either oral pirfenidone (a pyridine with anti-fibrotic activity) or nintedanib (tyrosine kinase inhibitor), oxygen therapy, pulmonary rehabilitation, or in some instances lung transplant [112]. Recently, prostacyclin analogs [103,113] and phosphoinositide 3-kinase (PI3K) pan inhibitors [114,115] have shown promise in models of PF with examples of both classes of drugs being evaluated via the inhaled route of administration.

**Figure 4:**
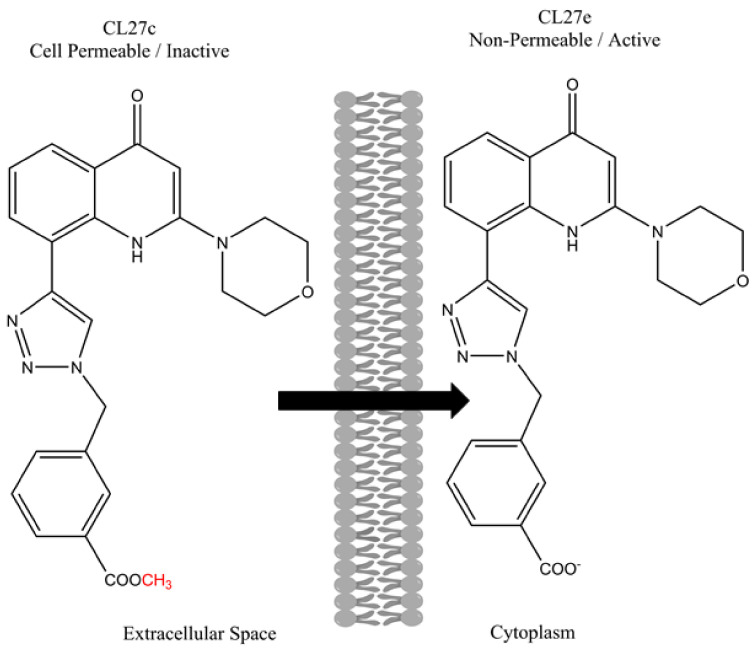
Chemical structures of the active compound CL27e and the corresponding methyl ester prodrug CL27c. Adapted from [119].

The PI3K inhibition strategy is interesting because PI3Ks are involved in a variety of biological processes related to inflammatory conditions such as PF, autoimmune disorders, and certain cancers [116,117]. Recently, Pirali et al. (2017) developed a novel PI3K inhibitor by synthesizing a small library of triazolylquinolones and screening them for PI3K inhibition [118]. Using in vitro PI3K inhibition assays, the authors identified a promising candidate termed CL27e. However, during cell based PI3K activity screening, CL27e failed to affect the PI3K signaling pathway. The authors hypothesized that the ionizable carboxylic acid increased the hydrophilicity of the compound and prevented it from crossing cell membranes and entering the cytoplasm. In other words, the compound could not reach the site of interest and required further modification. To enhance the cell permeation, the authors evaluated a series of ester-based prodrugs, identifying the methyl ester derivative CL27c as the lead candidate that was most effective at inhibiting the PI3K signaling pathway in vitro. The structures of both CL27c and CL27e are shown below in Figure 4.

**Figure 5:**
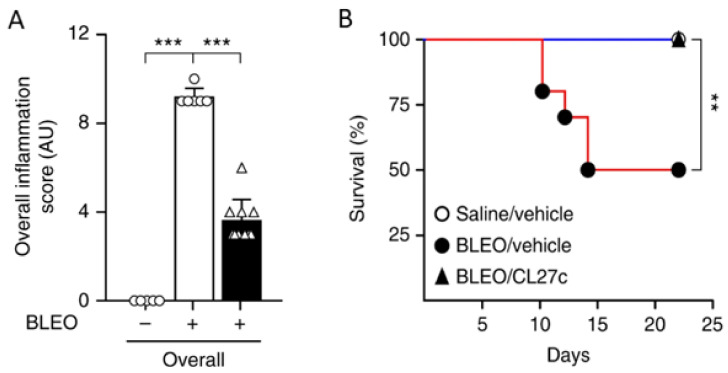
In a bleomycin-induced model of pulmonary fibrosis, treatment with CL27c results in reduced inflammation (**A**) and increased survival (**B**). Histopathologic scoring of inflammatory damage in lung sections derived from control (Bleo−) and Bleo-treated mice (Bleo+) with and without treatment with CL27c (black and white bars, respectively) (n = 5, 6, 10 independent experiments). *** *p* < 0.001 determined using Kruskal–Wallis followed by Dunn’s test. ** *p* < 0.01 determined using one-way ANOVA followed by Bonferroni post-hoc test. Adapted from [120]. Nature Communications, 2018.

In a follow-up publication, Campa et al. (2018) reported on how inhaled delivery of CL27c can improve lung function in rodent models of asthma and fibrosis [120]. Importantly, CL27c was delivered via nebulization to avoid unwanted on-target systemic toxicity observed for other PI3K inhibitors [121,122,123,124]. The results indicate that CL27c delivered via inhalation improves lung function in murine models of acute asthma and protects against bleomycin-induced pulmonary fibrosis. Specifically, inhaled CL27c resulted in reduced inflammation and improved survival rate in a bleomycin-induced model of pulmonary fibrosis (Figure 5).

In summary, if human clinical data replicates these preclinical findings, then the development of CL27c will demonstrate that the combination of prodrug modification that requires intracellular accumulation for release of the active compound, with targeted delivery to the lungs to reduce systemic on-target side effects observed for PI3K inhibitors, can be an effective strategy to overcome biological barriers associated with pulmonary delivery.

## 6. Inhaled Liposomal Ciprofloxacin for the Treatment of Non-Cystic Fibrosis Bronchiectasis (NCFBE)

NCFBE is characterized by a vicious cycle of infection and inflammation, which leads to structural damage to the airways and deterioration in lung function [125]. NCFBE patients with chronic lung infections have a decreased quality of life and greater morbidity. *Pseudomonas aeruginosa* (PA) lung infections in particular are associated with a seven times greater risk of hospitalization and three times greater mortality compared to NCFBE patients who are uninfected [126]. Thus, an effective inhaled antibiotic targeting PA lung infections in NCFBE patients may reduce the incidence of pulmonary exacerbations and improve morbidity and quality of life. While inhaled antibiotics have become a mainstay of therapy in CF, they have generally been unsuccessful in demonstrating clinical benefit in NCFBE and have been associated with an increased incidence of respiratory adverse events [127,128], bronchospasm [129,130] and drug withdrawals [127,128,129], compared to placebo. Thus, one of the key barriers to developing an effective inhaled antibiotic in NCFBE is to overcome their poor tolerability [131]. Additionally, most inhaled antibiotics are rapidly absorbed systemically after deposition in the lung, which may necessitate multiple administration events each day to ensure that the antibiotic concentrations remain above the pathogen’s minimum inhibitory concentration (MIC) [131,132]. In CF, inhaled tobramycin is labeled for twice-daily administration and aztreonam is administered three times a day. A sustained release formulation of an inhaled antibiotic, which slowly exposes the lung surface to the antibiotic over a 24-h period, thus has the potential to address both the tolerability and residence time barriers.

There are many possible formulation strategies that can provide a sustained release profile in the lung, but liposomes have emerged as a viable strategy for a diverse range of molecules, including antibiotics [69,133]. Robust and reproducible inhaled liposomal formulations can be manufactured utilizing lipids endogenous to the lung including phospholipids and cholesterol [134]. Inhalation and deposition of a biocompatible liposomal formulation on the surface of the airways is unlikely to cause local irritation or sudden perturbations to pH or osmolarity, factors which can lead to cough or bronchospasm [131,132]. A liposomal formulation of ciprofloxacin was thus developed with the goal to provide a sustained release profile in the lung following once-daily inhalation in NCFBE patients with PA lung infections [132].

The development of an inhaled liposomal formulation can be more challenging than the development of traditional inhaled nebulizer solutions and a number of hurdles may need to be addressed: reproducibility of manufacturing each liposome batch, stability of the formulation over its shelf-life, stability of the formulation to the aerosolization process, generation of an appropriate aerosol particle size distribution allowing deposition of an effective dose in the airways, and release of drug from the liposomes at an appropriate rate to maintain drug levels above the MIC until the next administration event [134].

**Figure 6:**
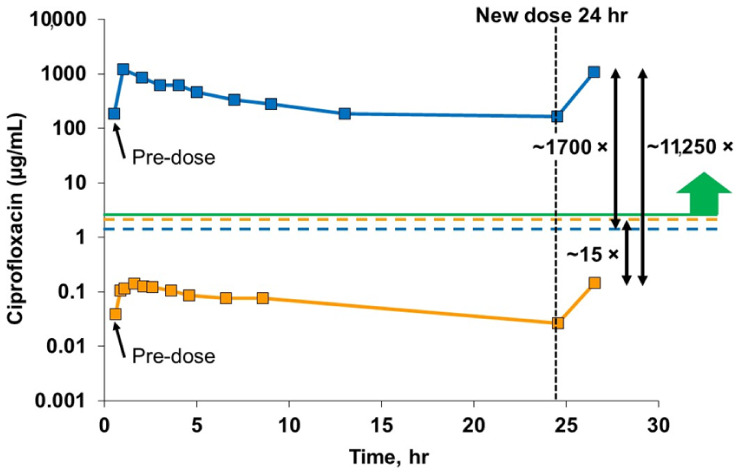
Ciprofloxacin concentrations in blood and sputum samples at steady state. These results cover a single dosing interval over a 24-h period from the Phase 3 open label extension. These data are at steady state achieved after about 4 days of dosing. The solid blue line shows the very high sputum concentrations of ciprofloxacin achieved with inhaled liposomal ciprofloxacin, three orders of magnitude above the MIC for PA (green line) throughout the 24-h dosing interval, and well above the Cmax for oral ciprofloxacin in sputum (dotted blue line). This solid orange line shows a far lower plasma concentration of ciprofloxacin for inhaled liposomal ciprofloxacin, one order of magnitude lower than the Cmax for oral ciprofloxacin (dotted orange line). The peak sputum concentration of ciprofloxacin for inhaled liposomal ciprofloxacin is over 10,000× higher than the peak plasma concentration. Adapted from [135]. World Bronchiectasis Conference, 2017.

The liposomal ciprofloxacin formulation that was taken into late-stage clinical trials was composed of cholesterol and hydrogenated soy phosphatidylcholine (HSPC) in unilamellar liposomes of 90 nm diameter [132]. This formulation retained its liposome morphology, particle size distribution and in vitro release profile after jet nebulization [132]. The phase 3 trial of inhaled liposomal ciprofloxacin provided PK data that validated the choice of the inhalation route to improve selectivity of the drug for the lung and the choice of the liposome formulation to increase the drug residence time in the lung [135] (Figure 6). Utilization of the inhalation route resulted in a 1700-fold higher peak drug concentration in the sputum compared to oral ciprofloxacin at a similar dose, and a 15-fold lower systemic drug concentration [135]. Consistent with the design thesis, the liposomal component of the formulation provided a sustained presence of drug in the lung over the 24-h period [135].

In the pooled phase 3 trials, inhaled liposomal ciprofloxacin resulted in a delay in the time to first exacerbation of 65 days, which did not reach statistical significance (HR = 0.82 and *p* = 0.074); however, the frequency of moderate to severe pulmonary exacerbations (PE) was significantly (*p* = 0.0001) reduced by 33% and the frequency of severe PEs was significantly (*p* = 0.014) reduced by 42% [136]. The prespecified quality of life metric did not demonstrate improvement in respiratory symptoms at the end of the 48-week trial compared to baseline [137]. However, because liposomal ciprofloxacin was administered in six cycles of 28 days on treatment followed by a 28-day drug holiday, a post-hoc analysis of the on-treatment periods demonstrated significant improvements in respiratory symptoms and declines in CFUs that correlated with improvements in symptoms (*p* < 0.0001) [137]. Following FDA review of clinical trial data, a complete response letter was issued and liposomal ciprofloxacin remains unapproved. While the complete response letter provides a reminder that preclinical success may not always translate to the clinic, the development work and preclinical data surrounding inhaled liposomal ciprofloxacin does validate the use of liposomes as a formulation tool to help overcome biological barriers, specifically in terms of improved patient tolerability and drug residence time in the lung with reduced systemic exposure.

## 7. Inhaled Liposomal Amikacin for the Treatment of Non-Tuberculous Mycobacteria (NTM)

NTM are opportunistic pathogens that are ubiquitous in the environment and can cause pulmonary infections in patients who typically also have underlying respiratory conditions [138,139]. When established, NTM pulmonary infection presents as NTM lung disease. Among mycobacterial species that can cause NTM lung disease, the *Mycobacterium avium* complex (MAC) predominates, while *Mycobacterium abscessus* (Mab) is less common but more pathogenic [138].

NTM can exist extracellularly, in a biofilm form, or intracellularly within macrophages and other cells. NTM biofilms are found in sputum samples and in explanted lungs from CF patients infected with Mab [140]. NTM species can also effectively survive and persist intracellularly evading macrophage’s killing mechanisms [141,142] and NTM infections have been found in clinical samples with *M. avium* detected inside peripheral blood leukocytes and bone marrow aspirate [143,144]. Therefore, delivering an effective dose of antibiotic into cells infected with NTM should be an essential component of treatment.

NTM pathogens in planktonic form are sensitive to aminoglycoside antibiotics such as amikacin [145] that demonstrate both inhibitory and bactericidal effects. However, aminoglycoside antibiotics accumulate poorly in cells due to their highly hydrophilic nature. This limits their effectiveness against intracellular infections. By packaging amikacin into liposomes, targeted delivery into the intracellular compartment of macrophages was achieved with improved amikacin activity against intracellular *M. avium* infections [146].

Efficient NTM lung disease treatment requires delivery of high amounts of amikacin to the lung while keeping systemic concentrations low to avoid nephro- and oto-toxicities [147]. Inhalation delivery of liposomal amikacin directly into the lungs may address this problem, but this approach faces three major delivery challenges: (1) efficient delivery of mostly intact liposomes to the lungs; (2) effective distribution of the intact liposomes throughout the lungs; and (3) penetration into biofilms and macrophages to reach the sites of infection. Amikacin liposome inhalation suspension (ALIS), also referred to as Arikayce, or liposomal amikacin for inhalation (LAI), was developed to overcome these challenges and improve the treatment of NTM lung disease.

**Figure 7:**
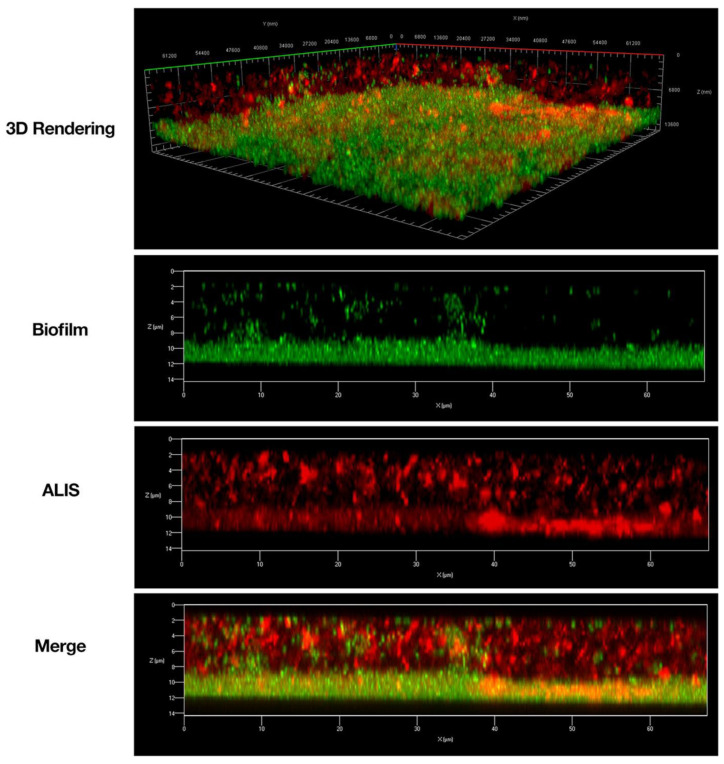
Amikacin liposome inhalation suspension (ALIS) penetrated *Mycobacterium avium* biofilms. Biofilms (strain A5) were established for 7 days in 2-well chamber slides, treated with 512 μg/mL of AF657-labeled ALIS (red) for 4 h, fixed, stained with Syto9 (green), and imaged with a Zeiss LSM 780 confocal scanning microscope (630× magnification). Adapted from [149]. Frontiers, 2018.

Liposomal amikacin (ALIS) was tested in in vitro and in vivo preclinical studies to assess whether it was an improvement over amikacin alone with respect to penetration into biofilms and macrophages. In an in vitro study, ALIS liposomes penetrated readily into PA biofilms and through a layer of CF patient mucus in 30 min, that larger (1 µm) fluorescent beads were not able to penetrate [148]. ALIS also penetrated *M. avium* biofilms [149] over a period of 4 h (Figure 7). In this study, mycobacteria biofilm were composed of a dense layer on the slide surface, with more diffuse bacteria and extracellular biofilm components present above. Liposomes could be seen distributed throughout the biofilm, demonstrating that the liposomes penetrated through the extracellular components and reached the cell dense region, suggesting that they may perform well in vivo.

**Figure 8:**
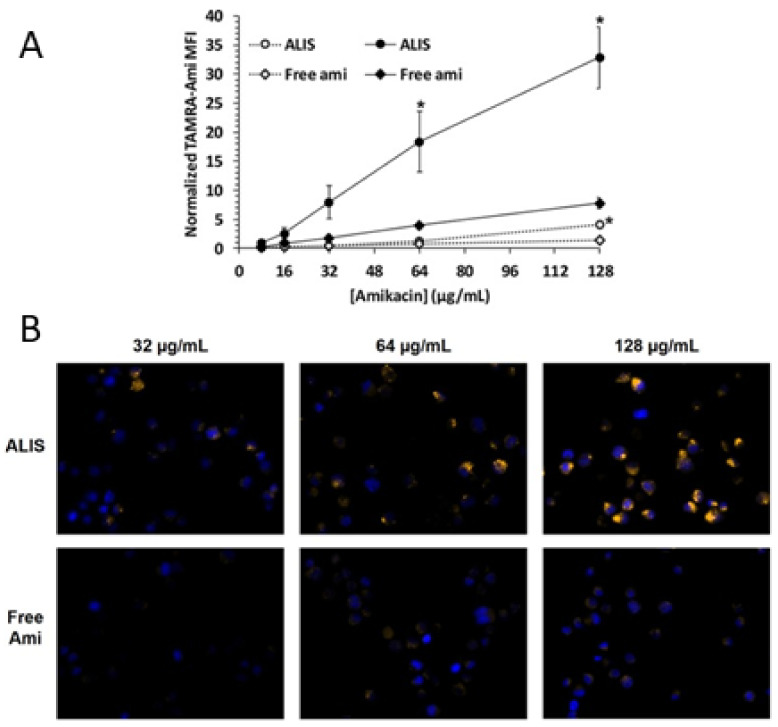
Liposomal and free amikacin uptake into human macrophages. Macrophages were exposed to increasing concentrations of either ALIS or free amikacin (with addition of 0.44% tetramethyl rhodamine (TAMRA) conjugated amikacin) for 4 h (gray symbols) or 24 h (black symbols). (**A**) normalized mean fluorescence intensity (MFI) at each concentration averaged from three independent experiments. (**B**) Visualization of liposomal and free amikacin uptake in human macrophages by fluorescence microscopy. TAMRA fluorescence was visualized by a Zeiss Axio fluorescence microscope (400× magnification) using constant settings for all experimental conditions. Yellow: TAMRA amikacin; Blue: DAPI-stained DNA. * *p* < 0.05 vs. free amikacin at the same concentration and timepoint. Adapted from [149]. Frontiers, 2018. Furthermore, tissue and pulmonary macrophage exposures were compared in vivo after the 96 mg/kg amikacin dose delivered by ALIS nebulization versus the 100 mg/kg amikacin dose given intravenously [149]. In pulmonary macrophages, maximum amikacin concentration (Cmax) after the ALIS dose was nearly 1.0 μg/μg protein and the total macrophage exposure over 24 h (AUC) was 17.8 μg*h/μg protein, 274-fold higher than the exposure following amikacin injection. Similarly, the BAL fluid and lung tissue exposures were 69.5- and 42.7-fold higher, respectively, after inhalation dosing of ALIS compared to intravenous dosing. Simultaneously, the systemic (blood plasma) exposure was 5-fold lower for ALIS than for amikacin injection.

The uptake of free or liposomal amikacin into human macrophages was quantified after incubation for 4 or 24 h [149]. Amikacin uptake was time dependent, with cells treated with ALIS containing significantly more amikacin than cells treated with the same concentration of free amikacin (Figure 8A) after 24 h. The amikacin accumulation in macrophages was up to 4-fold higher when treated with ALIS as compared to free amikacin, with meaningful differences between the groups exposed to drug concentrations of 64 and 128 μg/mL. Fluorescence microscopy images taken after 24-h incubation were consistent with the quantitative measurements: macrophages exposed to 32, 64, or 128 μg/mL of ALIS clearly exhibited bright TAMRA fluorescence (yellow) colocalized next to blue DAPI-stained cell nuclei, whereas TAMRA fluorescence was barely visible in cells incubated with free amikacin (Figure 8B).

During chronic daily administration of ALIS, lung alveolar macrophages may accumulate significant amounts of liposomal amikacin over time, thus raising the question of their possible effect on macrophage function. Therefore, an in vivo study was conducted to test the effect of continuous treatment of healthy rats by inhalation at a dose of 90 mg/kg over three 30-day treatment periods each followed by a 30-day recovery period [150]. Macrophages demonstrated accumulation of amikacin during treatment periods and nearly complete elimination during recovery periods. The 30-day dosing did not alter macrophage phagocytic activity, yeast killing function, or ability to release inflammatory mediators compared to the control group.

Having established that liposomal amikacin is superior to free amikacin with respect to macrophage uptake, the use of a sophisticated liposomal formulation like ALIS introduced a series of challenges that then had to be addressed, including the ability to nebulize the liposomal amikacin with consistent retention of its liposome properties. To ensure that any batch of liposomal amikacin that satisfied its release specification would provide consistent aerosol performance when used with an intended nebulizer device, in vitro studies were conducted to characterize the product performance [151]. In those studies, nebulized ALIS was shown to maintain a consistent aerosol delivery rate of 0.6 mL/min, aerosol emitted dose of 500 mg amikacin, aerosol fine particle dose of 260 mg amikacin, mean liposome vesicle size between 269–296 nm, and reproducibly generated 35% free amikacin and 65% encapsulated amikacin [151]. Furthermore, in a separate study, aerosol droplets of ALIS were segregated by size and collected in an impactor for subsequent evaluation [152]. In that study, the six aerosol size fractions ranging between 1.1 and 9 µm all maintained equivalent ratios of drug to lipid, percentage of encapsulated and free amikacin, and liposome size [152]. These in vitro experiments provided confidence that nebulized ALIS would perform reproducibly during clinical testing.

In healthy animals dosed with ALIS by inhalation, amikacin was distributed evenly throughout the lungs after single and multiple doses, with equal amikacin concentrations in all lobes of both lungs [150]. In clinical studies, when delivered by inhalation through nebulization, 43% of the ALIS nominal liposome dose was deposited in the lungs of NTM-LD patients [153]. Inhaled ALIS distributed throughout the whole lungs in both healthy volunteers [154] and patients with NTM-LD [153]; the ratio of distribution to central and peripheral lung regions was approximately 1.6–2.0 and more than 50% of the deposited dose was detectable in the lung 24 h post-dose.

Overall, the data demonstrate that nebulization of ALIS results in amikacin delivery to pulmonary macrophages, airways, and lung tissue better than free amikacin given by either inhalation or intravenous administration. This mechanism of improved delivery into pulmonary macrophages and retention within airways and lung tissue has been shown to effectively treat refractory NTM-LD in clinical trials and represents a promising new therapy for patients [155].

ALIS has been shown to effectively target NTM in both preclinical studies and clinical trials. In mice with pulmonary *M. avium* infections, inhalation administration of ALIS lowered viable mycobacteria in the lungs by more than 2 log units [156]. A Phase II trial of patients with refractory NTM-LD demonstrated that ALIS increased the proportion of patients who achieved negative sputum cultures compared with placebo, and the time to first negative sputum culture was shorter with ALIS treatment versus placebo [157]. Based on full results from Phase III studies, ALIS met the primary endpoint by demonstrating that the addition of ALIS to guideline-based therapy eliminated evidence of NTM-LD caused by MAC in sputum by month 6 in a greater proportion of patients than guideline-based therapy alone [155,158,159]. Currently, ALIS is approved in the US, EU, and JPN as ARIKAYCE^®^ and is indicated for the treatment of *Mycobacterium avium* complex (MAC) lung disease as part of a combination antibacterial drug regimen for adult patients who have limited or no alternative treatment options [160].

## 8. Concluding Remarks

The selection of a prodrug chemistry or nanoparticle formulation, either individually or in combination, can be used to overcome biological barriers related to pulmonary delivery across a wide array of diseases. To develop an improved inhaled prostanoid therapy providing sustained exposure to non-irritating levels of TRE in the lung, a prodrug approach has shown feasibility in either an inhaled dry powder format or when combined with a lipid nanoparticle formulation for nebulization. Two inhaled liposomal products have been utilized to overcome the rapid clearance and subsequent systemic exposure of unencapsulated small molecule drugs. Inhaled liposomal ciprofloxacin was designed to also improve pulmonary tolerability and selectivity for the lung, while liposomal amikacin improved uptake into macrophages at the site of intracellular infection. Looking to the future, while there are many treatments that have been developed for patients with CF, and these have dramatically improved their quality of life and extended survival, a gene therapy that directly corrects the chloride ion channel defect provides the ultimate transformational promise for these patients. To correct the defects in the epithelial cells in the lung may require delivery via the inhalation route. This is also true for other genetic diseases that are manifested primarily in the lung like Primary Ciliary Dyskinesia, and Alpha 1 Antitrypsin Deficiency, where a huge unmet need still exists. Inhaled gene therapy medications will likely require sophisticated formulation strategies to protect and efficiently deliver their genetic cargo to the epithelial cells on the lung surface. The biological barriers become especially challenging to overcome because the genetic cargo is more susceptible to degradation than for traditional small molecular weight drugs and the molecules must remain intact prior to transport into the epithelial cells. The inhaled gene therapy products in development will likely build upon the learnings from the examples described in this review.

## Data Availability

All data available are reported in the article.

## References

[1] Anderson S., Atkins P., Bäckman P., Cipolla D., Clark A., Daviskas E., Disse B., Entcheva-Dimitrov P., Fuller R., Gonda I. (2022). Inhaled Medicines: Past, Present, and Future. Pharmacol. Rev..

[2] Cipolla D., Chan H.K. (2018). Current and Emerging Inhaled Therapies of Repositioned Drugs. Adv. Drug Deliv. Rev..

[3] Stein S.W., Thiel C.G. (2016). The History of Therapeutic Aerosols: A Chronological Review. J. Aerosol Med. Pulm. Drug Deliv..

[4] Finlay W.H. (2001). 6—Fluid dynamics in the respiratory tract. The Mechanics of Inhaled Pharmaceutical Aerosols.

[5] Finlay W.H. (2001). 7—Particle deposition in the respiratory tract. The Mechanics of Inhaled Pharmaceutical Aerosols.

[6] Roche N., Scheuch G., Pritchard J.N., Nopitsch-Mai C., Lakhani D.A., Saluja B., Jamieson J., Dundon A., Wallace R., Holmes S. (2016). Patient Focus and Regulatory Considerations for Inhalation Device Design: Report from the 2015 IPAC-RS/ISAM Workshop. J. Aerosol Med. Pulm. Drug Deliv..

[7] Dundon A., Cipolla D., Mitchell J., Lyapustina S. (2020). Reflections on Digital Health Tools for Respiratory Applications. J. Aerosol Med. Pulm. Drug Deliv..

[8] Patton J.S., Brain J.D., Davies L.A., Fiegel J., Gumbleton M., Kim K.-J., Sakagami M., Vanbever R., Ehrhardt C. (2010). The Particle has Landed—Characterizing the Fate of Inhaled Pharmaceuticals. J. Aerosol Med. Pulm. Drug Deliv..

[9] Canning B.J., Farmer D.G., Mori N. (2006). Mechanistic studies of acid-evoked coughing in anesthetized guinea pigs. Am. J. Physiol. Regul. Integr. Comp. Physiol..

[10] Lowry R.H., Wood A.M., Higenbottam T.W. (1988). Effects of pH and osmolarity on aerosol-induced cough in normal volunteers. Clin. Sci..

[11] Higenbottam T. (1987). The mechanism of aerosol-induced bronchoconstriction. Bull. Eur. Physiopathol. Respir..

[12] Grace M., Birrell M.A., Dubuis E., Maher S.A., Belvisi M.G. (2012). Transient receptor potential channels mediate the tussive response to prostaglandin E2 and bradykinin. Thorax.

[13] Birrell M.A., Belvisi M.G., Grace M., Sadofsky L., Faruqi S., Hele D.J., Maher S.A., Freund-Michel V., Morice A.H. (2009). TRPA1 agonists evoke coughing in guinea pig and human volunteers. Am. J. Respir. Crit. Care Med..

[14] Eschenbacher W.L., Boushey H.A., Sheppard D. (1984). Alteration in osmolarity of inhaled aerosols cause bronchoconstriction and cough, but absence of a permeant anion causes cough alone. Am. Rev. Respir. Dis..

[15] Geller D.E., Nasr S.Z., Piggott S., He E., Angyalosi G., Higgins M. (2014). Tobramycin inhalation powder in cystic fibrosis patients: Response by age group. Respir. Care.

[16] Kastelik J.A., Thompson R.H., Aziz I., Ojoo J.C., Redington A.E., Morice A.H. (2002). Sex-related differences in cough reflex sensitivity in patients with chronic cough. Am. J. Respir. Crit. Care Med..

[17] Ebihara S., Ebihara T., Kohzuki M. (2012). Effect of aging on cough and swallowing reflexes: Implications for preventing aspiration pneumonia. Lung.

[18] Newnham D.M., Hamilton S.J. (1997). Sensitivity of the cough reflex in young and elderly subjects. Age Ageing.

[19] Sahakijpijarn S., Smyth H.D.C., Miller D.P., Weers J.G. (2020). Post-inhalation cough with therapeutic aerosols: Formulation considerations. Adv. Drug Deliv. Rev..

[20] Winkler J., Hochhaus G., Derendorf H. (2004). How the lung handles drugs: Pharmacokinetics and pharmacodynamics of inhaled corticosteroids. Proc. Am. Thorac. Soc..

[21] Biddiscombe M., Meah S., Barnes P., Usmani O. (2016). Drug particle size and lung deposition in COPD. Eur. Respir. J..

[22] Sangwan S., Agosti J.M., Bauer L.A., Otulana B.A., Morishige R.J., Cipolla D.C., Blanchard J.D., Smaldone G.C. (2001). Aerosolized protein delivery in asthma: Gamma camera analysis of regional deposition and perfusion. J. Aerosol Med..

[23] Brandsma C.A., Van den Berge M., Hackett T.L., Brusselle G., Timens W. (2020). Recent advances in chronic obstructive pulmonary disease pathogenesis: From disease mechanisms to precision medicine. J. Pathol..

[24] Boucher R.C. (2019). Muco-Obstructive Lung Diseases. N. Engl. J. Med..

[25] Matthay M.A., Zemans R.L., Zimmerman G.A., Arabi Y.M., Beitler J.R., Mercat A., Herridge M., Randolph A.G., Calfee C.S. (2019). Acute respiratory distress syndrome. Nat. Rev. Dis. Primers.

[26] Fahy J.V., Dickey B.F. (2010). Airway mucus function and dysfunction. N. Engl. J. Med..

[27] Sims D.E., Horne M.M. (1997). Heterogeneity of the composition and thickness of tracheal mucus in rats. Am. J. Physiol..

[28] Duncan G.A., Jung J., Hanes J., Suk J.S. (2016). The Mucus Barrier to Inhaled Gene Therapy. Mol. Ther..

[29] Lamblin G., Degroote S., Perini J.M., Delmotte P., Scharfman A., Davril M., Lo-Guidice J.M., Houdret N., Dumur V., Klein A. (2001). Human airway mucin glycosylation: A combinatory of carbohydrate determinants which vary in cystic fibrosis. Glycoconj. J..

[30] Matsui H., Randell S.H., Peretti S.W., Davis C.W., Boucher R.C. (1998). Coordinated clearance of periciliary liquid and mucus from airway surfaces. J. Clin. Investig..

[31] Donaldson S.H., Corcoran T.E., Laube B.L., Bennett W.D. (2007). Mucociliary clearance as an outcome measure for cystic fibrosis clinical research. Proc. Am. Thorac. Soc..

[32] Patton J.S., Fishburn C.S., Weers J.G. (2004). The lungs as a portal of entry for systemic drug delivery. Proc. Am. Thorac. Soc..

[33] Serisier D.J., Carroll M.P., Shute J.K., Young S.A. (2009). Macrorheology of cystic fibrosis, chronic obstructive pulmonary disease & normal sputum. Respir. Res..

[34] Takano M., Kawami M., Aoki A., Yumoto R. (2015). Receptor-mediated endocytosis of macromolecules and strategy to enhance their transport in alveolar epithelial cells. Exp. Opin. Drug Deliv..

[35] Palaniyandi S., Tomei E., Li Z., Conrad D.H., Zhu X. (2011). CD23-Dependent Transcytosis of IgE and Immune Complex across the Polarized Human Respiratory Epithelial Cells. J. Immunol..

[36] Amasheh S., Meiri N., Gitter A.H., Schöneberg T., Mankertz J., Schulzke J.D., Fromm M. (2002). Claudin-2 expression induces cation-selective channels in tight junctions of epithelial cells. J. Cell. Sci..

[37] Watson C.J., Rowland M., Warhurst G. (2001). Functional modeling of tight junctions in intestinal cell monolayers using polyethylene glycol oligomers. Am. J. Physiol. Cell Physiol..

[38] Hasegawa H., Fujita H., Katoh H., Aoki J., Nakamura K., Ichikawa A., Negishi M. (1999). Opposite regulation of transepithelial electrical resistance and paracellular permeability by Rho in Madin-Darby canine kidney cells. J. Biol. Chem..

[39] LaFemina M.J., Sutherland K.M., Bentley T., Gonzales L.W., Allen L., Chapin C.J., Rokkam D., Sweerus K.A., Dobbs L.G., Ballard P.L. (2014). Claudin-18 deficiency results in alveolar barrier dysfunction and impaired alveologenesis in mice. Am. J. Respir. Cell Mol. Biol..

[40] Sidhaye V.K., Chau E., Breysse P.N., King L.S. (2011). Septin-2 mediates airway epithelial barrier function in physiologic and pathologic conditions. Am. J. Respir. Cell Mol. Biol..

[41] Wittekindt O.H. (2017). Tight junctions in pulmonary epithelia during lung inflammation. Pflug. Arch..

[42] Soini Y. (2011). Claudins in lung diseases. Respir. Res..

[43] Yang Z.C., Yi M.J., Ran N., Wang C., Fu P., Feng X.Y., Xu L., Qu Z.H. (2013). Transforming growth factor-β1 induces bronchial epithelial cells to mesenchymal transition by activating the Snail pathway and promotes airway remodeling in asthma. Mol. Med. Rep..

[44] Cano A., Pérez-Moreno M.A., Rodrigo I., Locascio A., Blanco M.J., del Barrio M.G., Portillo F., Nieto M.A. (2000). The transcription factor snail controls epithelial-mesenchymal transitions by repressing E-cadherin expression. Nat. Cell Biol..

[45] Deshpande R., Zou C. (2020). *Pseudomonas Aeruginosa* Induced Cell Death in Acute Lung Injury and Acute Respiratory Distress Syndrome. Int. J. Mol. Sci..

[46] Guinee D., Brambilla E., Fleming M., Hayashi T., Rahn M., Koss M., Ferrans V., Travis W. (1997). The potential role of BAX and BCL-2 expression in diffuse alveolar damage. Am. J. Pathol..

[47] Bardales R.H., Xie S.S., Schaefer R.F., Hsu S.M. (1996). Apoptosis is a major pathway responsible for the resolution of type II pneumocytes in acute lung injury. Am. J. Pathol..

[48] Lehnert B.E., Valdez Y.E., Holland L.M. (1985). Pulmonary macrophages: Alveolar and interstitial populations. Exp. Lung Res..

[49] Hume P.S., Gibbings S.L., Jakubzick C.V., Tuder R.M., Curran-Everett D., Henson P.M., Smith B.J., Janssen W.J. (2020). Localization of Macrophages in the Human Lung via Design-based Stereology. Am. J. Respir. Crit. Care Med..

[50] Crapo J.D., Barry B.E., Gehr P., Bachofen M., Weibel E.R. (1982). Cell number and cell characteristics of the normal human lung. Am. Rev. Respir. Dis..

[51] Mosser D.M., Edwards J.P. (2008). Exploring the full spectrum of macrophage activation. Nat. Rev. Immunol..

[52] Martinez F.O., Gordon S. (2014). The M1 and M2 paradigm of macrophage activation: Time for reassessment. F1000Prime Rep..

[53] Dramé M., Buchrieser C., Escoll P. (2020). Danger-associated metabolic modifications during bacterial infection of macrophages. Int. Immunol..

[54] Pizarro-Cerdá J., Moreno E., Desjardins M., Gorvel J.P. (1997). When intracellular pathogens invade the frontiers of cell biology and immunology. Histol. Histopathol..

[55] Tanner L., Mashabela G.T., Omollo C.C., de Wet T.J., Parkinson C.J., Warner D.F., Haynes R.K., Wiesner L. (2021). Intracellular Accumulation of Novel and Clinically Used TB Drugs Potentiates Intracellular Synergy. Microbiol. Spectr..

[56] Barcia-Macay M., Seral C., Mingeot-Leclercq M.P., Tulkens P.M., Van Bambeke F. (2006). Pharmacodynamic evaluation of the intracellular activities of antibiotics against *Staphylococcus aureus* in a model of THP-1 macrophages. Antimicrob. Agents Chemother..

[57] Van Bambeke F., Carryn S., Seral C., Chanteux H., Tyteca D., Mingeot-Leclercq M.P., Tulkens P.M. (2004). Cellular pharmacokinetics and pharmacodynamics of the glycopeptide antibiotic oritavancin (LY333328) in a model of J774 mouse macrophages. Antimicrob. Agents Chemother..

[58] Plaunt A.J., Rose S.J., Kang J.Y., Chen K.J., LaSala D., Heckler R.P., Dorfman A., Smith B.T., Chun D., Viramontes V. (2021). Development and Preclinical Evaluation of New Inhaled Lipoglycopeptides for the Treatment of Persistent Pulmonary Methicillin-Resistant *Staphylococcus aureus* Infections. Antimicrob. Agents Chemother..

[59] Briones E., Colino C.I., Lanao J.M. (2008). Delivery systems to increase the selectivity of antibiotics in phagocytic cells. J. Control. Release.

[60] Pinto-Alphandary H., Andremont A., Couvreur P. (2000). Targeted delivery of antibiotics using liposomes and nanoparticles: Research and applications. Int. J. Antimicrob. Agents.

[61] Deng Y., Zhang X., Shen H., He Q., Wu Z., Liao W., Yuan M. (2020). Application of the Nano-Drug Delivery System in Treatment of Cardiovascular Diseases. Front. Bioeng. Biotechnol..

[62] Chenthamara D., Subramaniam S., Ramakrishnan S.G., Krishnaswamy S., Essa M.M., Lin F.-H., Qoronfleh M.W. (2019). Therapeutic efficacy of nanoparticles and routes of administration. Biomater. Res..

[63] Wang A.Z., Langer R., Farokhzad O.C. (2012). Nanoparticle delivery of cancer drugs. Annu. Rev. Med..

[64] Wilczewska A.Z., Niemirowicz K., Markiewicz K.H., Car H. (2012). Nanoparticles as drug delivery systems. Pharmacol. Rep..

[65] Luo M.-X., Hua S., Shang Q.-Y. (2021). Application of nanotechnology in drug delivery systems for respiratory diseases (Review). Mol. Med. Rep..

[66] Xie H., Liu C., Gao J., Shi J., Ni F., Luo X., He Y., Ren G., Luo Z. (2021). Fabrication of Zein-Lecithin-EGCG complex nanoparticles: Characterization, controlled release in simulated gastrointestinal digestion. Food Chem..

[67] Franco P., Reverchon E., De Marco I. (2018). Zein/diclofenac sodium coprecipitation at micrometric and nanometric range by supercritical antisolvent processing. J. CO_2_ Util..

[68] Li T., Cipolla D., Rades T., Boyd B.J. (2018). Drug nanocrystallisation within liposomes. J. Control. Release.

[69] Cipolla D., Shekunov B., Blanchard J., Hickey A. (2014). Lipid-based carriers for pulmonary products: Preclinical development and case studies in humans. Adv. Drug. Deliv. Rev..

[70] Chan H.-K., Kwok P.C.L. (2011). Production methods for nanodrug particles using the bottom-up approach. Adv. Drug Deliv. Rev..

[71] Khatib I., Chow M.Y.T., Ruan J., Cipolla D., Chan H.-K. (2021). Modeling of a spray drying method to produce ciprofloxacin nanocrystals inside the liposomes utilizing a response surface methodology: Box-Behnken experimental design. Int. J. Pharm..

[72] Leung S.S.Y., Wong J., Guerra H.V., Samnick K., Prud’homme R.K., Chan H.-K. (2017). Porous mannitol carrier for pulmonary delivery of cyclosporine A nanoparticles. AAPS J..

[73] Cipolla D., Wu H., Gonda I., Chan H.-K. (2014). Aerosol Performance and Long-Term Stability of Surfactant-Associated Liposomal Ciprofloxacin Formulations with Modified Encapsulation and Release Properties. AAPS PharmSciTech.

[74] Weers J.G., Bell J., Chan H.K., Cipolla D., Dunbar C., Hickey A.J., Smith I.J. (2010). Pulmonary Formulations: What Remains to be Done?. J. Aerosol Med. Pulm. Drug Deliv..

[75] Abdifetah O., Na-Bangchang K. (2019). Pharmacokinetic studies of nanoparticles as a delivery system for conventional drugs and herb-derived compounds for cancer therapy: A systematic review. Int. J. Nanomed..

[76] Pramanik S., Mohanto S., Manne R., Rajendran R.R., Deepak A., Edapully S.J., Patil T., Katari O. (2021). Nanoparticle-Based Drug Delivery System: The Magic Bullet for the Treatment of Chronic Pulmonary Diseases. Mol. Pharm..

[77] Rautio J., Meanwell N.A., Di L., Hageman M.J. (2018). The expanding role of prodrugs in contemporary drug design and development. Nat. Rev. Drug Discov..

[78] Hsieh P.W., Hung C.F., Fang J.Y. (2009). Current prodrug design for drug discovery. Curr. Pharm. Des..

[79] Chapman R.W., Corboz M.R., Malinin V.S., Plaunt A.J., Konicek D.M., Li Z., Perkins W.R. (2020). An overview of the biology of a long-acting inhaled treprostinil prodrug. Pulm. Pharmacol. Ther..

[80] Gessler T., Seeger W., Schmehl T. (2011). The potential for inhaled treprostinil in the treatment of pulmonary arterial hypertension. Ther. Adv. Respir. Dis..

[81] Nadler S.T., Edelman J.D. (2010). Inhaled treprostinil and pulmonary arterial hypertension. Vasc. Health Risk Manag..

[82] St Croix C.M., Steinhorn R.H. (2016). New Thoughts about the Origin of Plexiform Lesions. Am. J. Respir. Crit. Care Med..

[83] Vonk Noordegraaf A., Galiè N. (2011). The role of the right ventricle in pulmonary arterial hypertension. Eur. Respir. Rev..

[84] Abe K., Toba M., Alzoubi A., Ito M., Fagan K.A., Cool C.D., Voelkel N.F., McMurtry I.F., Oka M. (2010). Formation of plexiform lesions in experimental severe pulmonary arterial hypertension. Circulation.

[85] Keshavarz A., Kadry H., Alobaida A., Ahsan F. (2020). Newer approaches and novel drugs for inhalational therapy for pulmonary arterial hypertension. Exp. Opin. Drug Deliv..

[86] Frumkin L.R. (2012). The pharmacological treatment of pulmonary arterial hypertension. Pharmacol. Rev..

[87] Channick R.N., Voswinckel R., Rubin L.J. (2012). Inhaled treprostinil: A therapeutic review. Drug. Des. Devel. Ther..

[88] Ruan C.-H., Dixon R.A.F., Willerson J.T., Ruan K.-H. (2010). Prostacyclin therapy for pulmonary arterial hypertension. Tex. Heart Inst. J..

[89] (2014). Tyvaso® (Treprostinil) [Package Insert].

[90] Kan P., Chen K.-J., Hsu C.-F., Lin Y.-F. (2018). Inhaled liposomal iloprost shows high drug encapsulation, extended release profile and potentials of improving patient compliance. Eur. Respir. J..

[91] Jain P.P., Leber R., Nagaraj C., Leitinger G., Lehofer B., Olschewski H., Olschewski A., Prassl R., Marsh L.M. (2014). Liposomal nanoparticles encapsulating iloprost exhibit enhanced vasodilation in pulmonary arteries. Int. J. Nanomed..

[92] Kleemann E., Schmehl T., Gessler T., Bakowsky U., Kissel T., Seeger W. (2007). Iloprost-containing liposomes for aerosol application in pulmonary arterial hypertension: Formulation aspects and stability. Pharm. Res..

[93] Kan P., Chen K.-J., Pan C. (2020). Inhaled liposomal treprostinil (L606) shows extended release in healthy volunteer, as well as prolongs pharmacological effect in hypoxia-induced rat. Eur. Respir. J..

[94] Kan P., Chen K.J., Pan C. Comparative Pharmacokinetics Between Tyvaso(R) and L606, Extended-Release Formulation of Treprostinil for Inhalation Therapy. Proceedings of the American Thoracic Society.

[95] Nakamura K., Akagi S., Ejiri K., Yoshida M., Miyoshi T., Toh N., Nakagawa K., Takaya Y., Matsubara H., Ito H. (2019). Current Treatment Strategies and Nanoparticle-Mediated Drug Delivery Systems for Pulmonary Arterial Hypertension. Int. J. Mol. Sci..

[96] Segura-Ibarra V., Wu S., Hassan N., Moran-Guerrero J.A., Ferrari M., Guha A., Karmouty-Quintana H., Blanco E. (2018). Nanotherapeutics for Treatment of Pulmonary Arterial Hypertension. Front. Physiol..

[97] Roscigno R.F., Vaughn T., Parsley E., Hunt T., Eldon M.A., Rubin L.J. (2021). Comparative bioavailability of inhaled treprostinil administered as LIQ861 and Tyvaso^®^ in healthy subjects. Vascul. Pharmacol..

[98] Roscigno R., Vaughn T., Anderson S., Wargin W., Hunt T., Hill N.S. (2020). Pharmacokinetics and tolerability of LIQ861, a novel dry-powder formulation of treprostinil. Pulm. Circ..

[99] Leifer F.G., Konicek D.M., Chen K.-J., Plaunt A.J., Salvail D., Laurent C.E., Corboz M.R., Li Z., Chapman R.W., Perkins W.R. (2018). Inhaled Treprostinil-Prodrug Lipid Nanoparticle Formulations Provide Long-Acting Pulmonary Vasodilation. Drug Res..

[100] Ismat F.A., Usansky H., Dhar Murthy S., Zou J., Teper A. (2021). Safety, tolerability, and pharmacokinetics (PK) of treprostinil palmitil inhalation powder (TPIP): A phase 1, randomised, double-blind, single- and multiple-dose study. Eur. Heart J..

[101] Corboz M.R., Li Z., Malinin V., Plaunt A.J., Konicek D.M., Leifer F.G., Chen K.J., Laurent C.E., Yin H., Biernat M.C. (2017). Preclinical Pharmacology and Pharmacokinetics of Inhaled Hexadecyl-Treprostinil (C16TR), a Pulmonary Vasodilator Prodrug. J. Pharmacol. Exp. Ther..

[102] Chapman R.W., Li Z., Corboz M.R., Gauani H., Plaunt A.J., Konicek D.M., Leifer F.G., Laurent C.E., Yin H., Salvail D. (2018). Inhaled hexadecyl-treprostinil provides pulmonary vasodilator activity at significantly lower plasma concentrations than infused treprostinil. Pulm. Pharmacol. Ther..

[103] Corboz M.R., Zhang J., LaSala D., DiPetrillo K., Li Z., Malinin V., Brower J., Kuehl P.J., Barrett T.E., Perkins W.R. (2018). Therapeutic administration of inhaled INS1009, a treprostinil prodrug formulation, inhibits bleomycin-induced pulmonary fibrosis in rats. Pulm. Pharmacol. Ther..

[104] Han D., Fernandez C., Sullivan E., Xu D., Perkins W., Darwish M., Rubino C. (2016). Safety and pharmacokinetics study of a single ascending dose of C16TR for inhalation (INS1009). Eur. Respir. J..

[105] Corboz M.R., Plaunt A.J., Malinin V., Li Z., Gauani H., Chun D., Cipolla D., Perkins W., Chapman R.W. Beneficial Effects of Treprostinil Palmitil in a Sugen/Hypoxia Rat Model of Pulmonary Arterial Hypertension; A Comparison with Inhaled and Intravenous Treprostinil and Oral Selexipag. Proceedings of the American Thoracic Society.

[106] Corboz M.R., Plaunt A.J., Malinin V., Li Z., Gauani H., Chun D., Cipolla D., Perkins W.R., Chapman R.W. (2021). Treprostinil palmitil inhibits the hemodynamic and histopathological changes in the pulmonary vasculature and heart in an animal model of pulmonary arterial hypertension. Eur. J. Pharmacol..

[107] Plaunt A.J., Islam S., Macaluso T., Gauani H., Baker T., Chun D., Viramontes V., Chang C., Corboz M.R., Chapman R.W. (2021). Development and Characterization of Treprostinil Palmitil Inhalation Aerosol for the Investigational Treatment of Pulmonary Arterial Hypertension. Int. J. Mol. Sci..

[108] Chapman R.W., Corboz M.R., Fernandez C., Sullivan E., Stautberg A., Plaunt A.J., Konicek D.M., Malinin V., Li Z., Cipolla D. (2021). Characterisation of cough evoked by inhaled treprostinil and treprostinil palmitil. ERJ Open Res..

[109] Barratt S.L., Creamer A., Hayton C., Chaudhuri N. (2018). Idiopathic Pulmonary Fibrosis (IPF): An Overview. J. Clin. Med..

[110] Lederer D.J., Martinez F.J. (2018). Idiopathic Pulmonary Fibrosis. N. Engl. J. Med..

[111] Pardo A., Selman M. (2016). Lung Fibroblasts, Aging, and Idiopathic Pulmonary Fibrosis. Ann. Am. Thorac. Soc..

[112] Laporta Hernandez R., Aguilar Perez M., Lázaro Carrasco M.T., Ussetti Gil P. (2018). Lung Transplantation in Idiopathic Pulmonary Fibrosis. Med. Sci..

[113] Nikitopoulou I., Manitsopoulos N., Kotanidou A., Tian X., Petrovic A., Magkou C., Ninou I., Aidinis V., Schermuly R.T., Kosanovic D. (2019). Orotracheal treprostinil administration attenuates bleomycin-induced lung injury, vascular remodeling, and fibrosis in mice. Pulm. Circ..

[114] Hettiarachchi S.U., Li Y.H., Roy J., Zhang F., Puchulu-Campanella E., Lindeman S.D., Srinivasarao M., Tsoyi K., Liang X., Ayaub E.A. (2020). Targeted inhibition of PI3 kinase/mTOR specifically in fibrotic lung fibroblasts suppresses pulmonary fibrosis in experimental models. Sci. Transl. Med..

[115] Lukey P.T., Harrison S.A., Yang S., Man Y., Holman B.F., Rashidnasab A., Azzopardi G., Grayer M., Simpson J.K., Bareille P. (2019). A randomised, placebo-controlled study of omipalisib (PI3K/mTOR) in idiopathic pulmonary fibrosis. Eur. Respir. J..

[116] Stark A.-K., Sriskantharajah S., Hessel E.M., Okkenhaug K. (2015). PI3K inhibitors in inflammation, autoimmunity and cancer. Curr. Opin. Pharmacol..

[117] Vanhaesebroeck B., Vogt P.K., Rommel C. (2010). PI3K: From the bench to the clinic and back. Curr. Top. Microbiol. Immunol..

[118] Pirali T., Ciraolo E., Aprile S., Massarotti A., Berndt A., Griglio A., Serafini M., Mercalli V., Landoni C., Campa C.C. (2017). Identification of a Potent Phosphoinositide 3-Kinase Pan Inhibitor Displaying a Strategic Carboxylic Acid Group and Development of Its Prodrugs. ChemMedChem.

[119] (2018). Pesquisadores Sintetizam Fármaco Para Tratamento de Asma e Fibrose Pulmonar. https://ufmg.br/comunicacao/noticias/pesquisadores-sintetizam-farmaco-para-tratamento-de-asma-e-fibrose-pulmonar.

[120] Campa C.C., Silva R.L., Margaria J.P., Pirali T., Mattos M.S., Kraemer L.R., Reis D.C., Grosa G., Copperi F., Dalmarco E.M. (2018). Inhalation of the prodrug PI3K inhibitor CL27c improves lung function in asthma and fibrosis. Nat. Commun..

[121] Fruman D.A., Chiu H., Hopkins B.D., Bagrodia S., Cantley L.C., Abraham R.T. (2017). The PI3K Pathway in Human Disease. Cell.

[122] Kulkarni S., Sitaru C., Jakus Z., Anderson K.E., Damoulakis G., Davidson K., Hirose M., Juss J., Oxley D., Chessa T.A. (2011). PI3Kβ plays a critical role in neutrophil activation by immune complexes. Sci. Signal..

[123] Condliffe A.M., Davidson K., Anderson K.E., Ellson C.D., Crabbe T., Okkenhaug K., Vanhaesebroeck B., Turner M., Webb L., Wymann M.P. (2005). Sequential activation of class IB and class IA PI3K is important for the primed respiratory burst of human but not murine neutrophils. Blood.

[124] Berghausen E.M., Moeller F., Vantler M., Hirsch E., Baldus S., Rosenkranz R. (2019). P1938In vitro characterization of a novel PI 3-kinase inhibitor for growth factor-induced effects in pulmonary artery smooth muscle cells. Eur. Heart J..

[125] McShane P.J., Naureckas E.T., Tino G., Strek M.E. (2013). Non–Cystic Fibrosis Bronchiectasis. Am. J. Respir. Crit. Care Med..

[126] Finch S., McDonnell M.J., Abo-Leyah H., Aliberti S., Chalmers J.D. (2015). A Comprehensive Analysis of the Impact of Pseudomonas aeruginosa Colonization on Prognosis in Adult Bronchiectasis. Ann. Am. Thorac. Soc..

[127] Drobnic M.E., Suñé P., Montoro J.B., Ferrer A., Orriols R. (2005). Inhaled tobramycin in non-cystic fibrosis patients with bronchiectasis and chronic bronchial infection with *Pseudomonas aeruginosa*. Ann. Pharmacother..

[128] Barker A.F., Couch L., Fiel S.B., Gotfried M.H., Ilowite J., Meyer K.C., O’Donnell A., Sahn S.A., Smith L.J., Stewart J.O. (2000). Tobramycin Solution for Inhalation Reduces Sputum *Pseudomonas aeruginosa* Density in Bronchiectasis. Am. J. Respir. Crit. Care Med..

[129] Bilton D., Henig N., Morrissey B., Gotfried M. (2006). Addition of inhaled tobramycin to ciprofloxacin for acute exacerbations of *Pseudomonas aeruginosa* infection in adult bronchiectasis. Chest.

[130] Rubin B.K. (2008). Aerosolized Antibiotics for Non-Cystic Fibrosis Bronchiectasis. J. Aerosol Med. Pulm. Drug Deliv..

[131] Weers J. (2015). Inhaled antimicrobial therapy—Barriers to effective treatment. Adv. Drug. Deliv. Rev..

[132] Cipolla D., Blanchard J., Gonda I. (2016). Development of Liposomal Ciprofloxacin to Treat Lung Infections. Pharmaceutics.

[133] Cipolla D., Gonda I., Chan H.K. (2013). Liposomal formulations for inhalation. Ther. Deliv..

[134] Cipolla D., Kwok P.C.L., Chan H.K. (2017). Inhaled Liposomes. Advances in Pulmonary Delivery.

[135] Froehlich J., Cipolla D., DeSoyza A., Morrish G., Gonda I. Inhaled Liposomal Ciprofloxacin in Patients with Non-Cystic Fibrosis Bronchiectasis and Chronic *Pseudomonas aeruginosa* Infection: Pharmacokinetics of Once-Daily Inhaled ARD-3150. Proceedings of the 2nd WBE Conference.

[136] Haworth C.S., Bilton D., Chalmers J.D., Davis A.M., Froehlich J., Gonda I., Thompson B., Wanner A., O’Donnell A.E. (2019). Inhaled liposomal ciprofloxacin in patients with non-cystic fibrosis bronchiectasis and chronic lung infection with *Pseudomonas aeruginosa* (ORBIT-3 and ORBIT-4): Two phase 3, randomised controlled trials. Lancet Respir. Med..

[137] Chalmers J.D., Cipolla D., Thompson B., Davis A.M., O’Donnell A., Tino G., Gonda I., Haworth C., Froehlich J. (2020). Changes in respiratory symptoms during 48-week treatment with ARD-3150 (inhaled liposomal ciprofloxacin) in bronchiectasis: Results from the ORBIT-3 and -4 studies. Eru. Respir. J..

[138] Johnson M.M., Odell J.A. (2014). Nontuberculous mycobacterial pulmonary infections. J. Thorac. Dis..

[139] Van Ingen J., Kuijper E.J. (2014). Drug susceptibility testing of nontuberculous mycobacteria. Future Microbiol..

[140] Qvist T., Eickhardt S., Kragh K.N., Andersen C.B., Iversen M., Høiby N., Bjarnsholt T. (2015). Chronic pulmonary disease with *Mycobacterium abscessus* complex is a biofilm infection. Eur. Respir. J..

[141] Appelberg R. (2006). Pathogenesis of *Mycobacterium avium* infection: Typical responses to an atypical mycobacterium?. Immunol. Res..

[142] Awuh J.A., Flo T.H. (2017). Molecular basis of mycobacterial survival in macrophages. Cell. Mol. Life Sci..

[143] Graham B.S., Hinson M.V., Bennett S.R., Gregory D.W., Schaffner W. (1984). Acid-fast bacilli on buffy coat smears in the acquired immunodeficiency syndrome: A lesson from Hansen’s bacillus. South. Med. J..

[144] Moffie B.G., Krulder J.W., de Knijff J.C. (1989). Direct visualization of mycobacteria in blood culture. N. Engl. J. Med..

[145] Cowman S., Burns K., Benson S., Wilson R., Loebinger M.R. (2016). The antimicrobial susceptibility of non-tuberculous mycobacteria. J. Infect..

[146] Kesavalu L., Goldstein J.A., Debs R.J., Düzgünes N., Gangadharam P.R. (1990). Differential effects of free and liposome encapsulated amikacin on the survival of *Mycobacterium avium* complex in mouse peritoneal macrophages. Tubercle.

[147] Olivier K.N., Shaw P.A., Glaser T.S., Bhattacharyya D., Fleshner M., Brewer C.C., Zalewski C.K., Folio L.R., Siegelman J.R., Shallom S. (2014). Inhaled amikacin for treatment of refractory pulmonary nontuberculous mycobacterial disease. Ann. Am. Thorac. Soc..

[148] Meers P., Neville M., Malinin V., Scotto A.W., Sardaryan G., Kurumunda R., Mackinson C., James G., Fisher S., Perkins W.R. (2008). Biofilm penetration, triggered release and in vivo activity of inhaled liposomal amikacin in chronic *Pseudomonas aeruginosa* lung infections. J. Antimicrob. Chemother..

[149] Zhang J., Leifer F., Rose S., Chun D.Y., Thaisz J., Herr T., Nashed M., Joseph J., Perkins W.R., DiPetrillo K. (2018). Amikacin Liposome Inhalation Suspension (ALIS) Penetrates Non-tuberculous Mycobacterial Biofilms and Enhances Amikacin Uptake Into Macrophages. Front. Microbiol..

[150] Malinin V., Neville M., Eagle G., Gupta R., Perkins W.R. (2016). Pulmonary Deposition and Elimination of Liposomal Amikacin for Inhalation and Effect on Macrophage Function after Administration in Rats. Antimicrob. Agents Chemother..

[151] Li Z., Perkins W., Cipolla D. (2021). Robustness of aerosol delivery of amikacin liposome inhalation suspension using the eFlow^®^ Technology. Eur. J. Pharm. Biopharm..

[152] Li Z., Zhang Y., Wurtz W., Lee J.K., Malinin V.S., Durwas-Krishnan S., Meers P., Perkins W.R. (2008). Characterization of Nebulized Liposomal Amikacin (Arikace™) as a Function of Droplet Size. J. Aerosol Med. Pulm. Drug Deliv..

[153] Olivier K.N., Maas-Moreno R., Whatley M., Cheng K., Lee J.H., Fiorentino C., Shaffer R., Macdonald S., Gupta R., Corcoran T.E. (2016). Airway Deposition and Retention of Liposomal Amikacin for Inhalation in Patients with Pulmonary Nontuberculous Mycobacterial Disease. Am. J. Respir. Crit. Care Med..

[154] Weers J., Metzheiser B., Taylor G., Warren S., Meers P., Perkins W.R. (2009). A gamma scintigraphy study to investigate lung deposition and clearance of inhaled amikacin-loaded liposomes in healthy male volunteers. J. Aerosol Med. Pulm. Drug Deliv..

[155] Griffith D.E., Eagle G., Thomson R., Aksamit T.R., Hasegawa N., Morimoto K., Addrizzo-Harris D.J., O’Donnell A.E., Marras T.K., Flume P.A. (2018). Amikacin Liposome Inhalation Suspension for Treatment-Refractory Lung Disease Caused by *Mycobacterium avium* Complex (CONVERT). A Prospective, Open-Label, Randomized Study. Am. J. Respir. Crit. Care Med..

[156] Rose S.J., Neville M.E., Gupta R., Bermudez L.E. (2014). Delivery of aerosolized liposomal amikacin as a novel approach for the treatment of nontuberculous mycobacteria in an experimental model of pulmonary infection. PLoS ONE.

[157] Olivier K.N., Griffith D.E., Eagle G., McGinnis J.P., Micioni L., Liu K., Daley C.L., Winthrop K.L., Ruoss S., Addrizzo-Harris D.J. (2017). Randomized Trial of Liposomal Amikacin for Inhalation in Nontuberculous Mycobacterial Lung Disease. Am. J. Respir. Crit. Care Med..

[158] Griffith D.E., Thomson R., Flume P.A., Aksamit T.R., Field S.K., Addrizzo-Harris D.J., Morimoto K., Hoefsloot W., Mange K.C., Yuen D.W. (2021). Amikacin Liposome Inhalation Suspension for Refractory Mycobacterium avium Complex Lung Disease: Sustainability and Durability of Culture Conversion and Safety of Long-term Exposure. Chest.

[159] Winthrop K.L., Flume P.A., Thomson R., Mange K.C., Yuen D.W., Ciesielska M., Morimoto K., Ruoss S.J., Codecasa L.R., Yim J.-J. (2021). Amikacin Liposome Inhalation Suspension for Mycobacterium avium Complex Lung Disease: A 12-Month Open-Label Extension Clinical Trial. Ann. Am. Thorac. Soc..

[160] Arikayce® (Amikacin Liposome Inhalation Suspension), for Oral Inhalation Use [Package Insert]. https://www.arikayce.com/pdf/full_prescribing_information.pdf.

